# An Informal Education Intervention in Response to the Covid-19 Pandemic: Homework Mentorships in a Berlin Refugee Shelter

**DOI:** 10.1007/s42087-020-00161-3

**Published:** 2021-01-20

**Authors:** Courtney O’Connell, Luka Lucić

**Affiliations:** 1 Potsdam University of Applied Sciences, + stadt.menschen.berlin, Berlin, Germany; 2 Social Science and Cultural Studies, Pratt Institute, Brooklyn, NY USA

**Keywords:** Youth, Refugees, Migration, Education, Segregation, Germany

## Abstract

The Covid-19 pandemic and resulting damage is often portrayed in staggering numbers and statistics. This article offers, by contrast, a personal and qualitative account of employees, volunteers, and young residents at a refugee home in Berlin, Germany. Through the story of a boy who has spent the past 4 years in several of Berlin’s 84 remaining refugee accommodations, we examine the inequalities that already existed in Germany and how the pandemic has exacerbated them. To provide ample context, we critically assess the so-called *Welcome Classes* that children and teens have been attending since their arrival to the country in or around 2015 and argue that the segregation experienced at school mirrors the isolation from the host society that refugees and people seeking asylum are subjected to residentially. We then present an emergency response to school closures: A digital homework mentorship program designed to mitigate the heightened barriers to social interaction and access to education brought about by the pandemic. We explore the sociocultural theory underpinning the program, describe its methods, and offer a qualitative evaluation of the results. Finally, we discuss how the informal education intervention helps fill gaps in the system, offering an approach that can be used going forward to promote learning, social-emotional development, and inclusion of young people with migration and refugee backgrounds.

## Introduction

In March of 2020, while Berlin was paralyzed by the outbreak of Covid-19, Oduman was one of the 193 people living at his shelter for people seeking asylum, and one of the 44 residents who had been there since it opened over three years prior. Oduman is around 11 years old, but no one is sure of his exact age. He fled Afghanistan with his parents and older brother when he was around three years old. After some months of traveling by foot and a few months waiting in Turkey, they completed the treacherous boat ride across the Mediterranean Sea to Greece. From there, they made their way to Berlin and arrived in 2016.

**Fig. 1:**
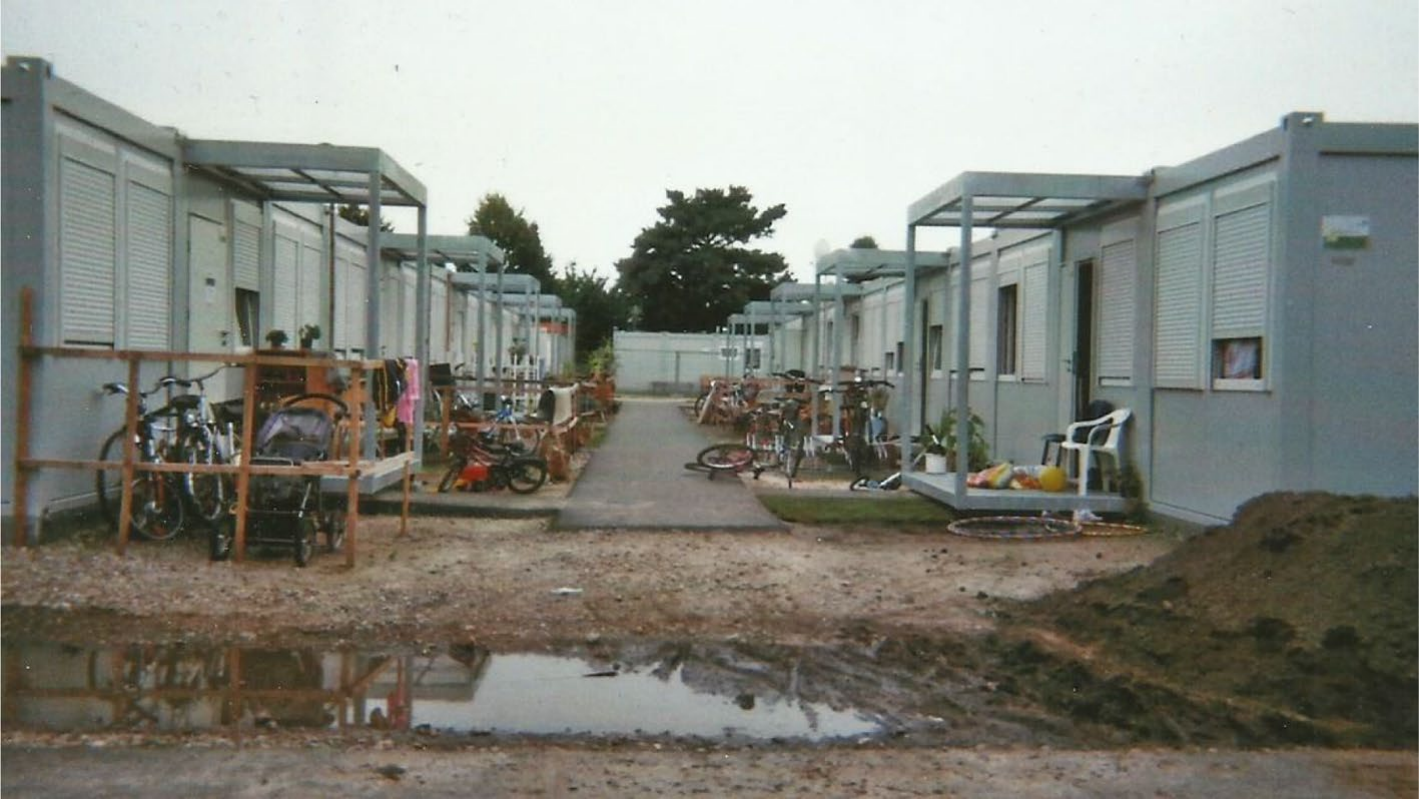
This photo is of a street in a “tempohomes” facility in Berlin. The picture was taken in 2017, shortly after people first moved into the containers. Construction was still underway while approximately 200 adults and 60 people under eighteen years old were residing on the premises.

While the German government invests a considerable amount of resources into the social integration of young people with refugee and migration backgrounds, in practice, the education and housing systems in place have structures built into them that prevent integration from taking place. Upon arrival, people seeking asylum are required to live in temporary accommodations, often for years on end and in geographically isolated locations, making interaction with German neighbors almost non-existent. For their first nine months in the city, Oduman and his family lived in several emergency shelters, usually converted sport halls. By November 2016, the first of Berlin’s 17 so-called “tempohomes” were opened (“Tempohomes FAQ”, n.d.). When the first busses full of people from emergency shelters—including Oduman and his family—arrived at the camp, they refused to exit the bus. They were under the impression that they would get to live in apartments, but these were not apartments; they were containers, like those found on construction sites (see Figure 1). Police had to force people out of the bus and into their new place of residence.

The seven containers on the premises hold 64 living units, each comprising two 13 square meter rooms, separated by a small kitchen and bathroom. People who are in Germany on their own share a room with another adult, and a total of four adults share a bathroom and kitchen. Families usually have a whole unit to themselves. There are two more containers within the camp: One for social services offices and laundry facilities and the other for community rooms, including a shared kitchen, a women’s room, an exercise room, a TV room, a study room, and a playroom for children.

After three years, the shelter, along with the other 16 “container villages” in Berlin, was supposed to close down. But at the particular location where Oduman and his family live—as well as in several others—the closing date has been repeatedly pushed back. At the time of writing, Oduman had lived in the same container for three years and six months—by best estimates from the time he was around seven years old. During his time at the shelter, Oduman has seen a lot. In 2018, he watched from the windows of the playroom as his 19-year-old brother was escorted out of the camp in handcuffs by police after they found drugs in his room. His brother was sent to live in a group home and never moved back in with his family. Oduman’s mother suffers from numerous physical and mental health ailments and his father speaks no German at all. Largely as a result of the circumstances in which he lives, Oduman is growing up with a complex network of roles and responsibilities that far exceed those of his German-born peers. When his brother left, he became the translator for virtually all family affairs and could regularly be found with his father in the social services container, discussing matters that most children his age would be shielded from.

Oduman’s case is not unique. As of June 2020, there were still 84 refugee shelters across Berlin, housing just over 22,000 people (Landesamt für Flüchtlingsangelegenheiten, 2020). While the task of overcoming spatial barriers features prominently during a refugee’s journey, it does not diminish with the arrival to the host society. Many of Berlin’s shelters are as geographically remote—and as socially isolated—as the one where Oduman lives, in a northeastern suburb on the very outskirts of the greater Berlin area. Fifteen kilometers from the city center in a quiet, suburban neighborhood, interactions with people not living in his shelter are few and far between.

Once inside the premises of the camp, surrounded by a fence and manned by security guards, life is defined by a lack of space. In a survey conducted by the Federal Association of the Psychosocial Centers for Refugees and Victims of Torture (BAfF e.V) in 2019—a year before the onset of the pandemic—residents of refugee shelters specifically described the absence of safe spaces for their children. Their interviews highlight that young people living in shelters had almost no personal space—for rest, learning, or play. Fear and threatening situations were part of everyday life across these large mass residential accommodations. “Nobody can sleep well here. It is always loud at night, someone is talking everywhere, children scream all night.[…] You always have to expect conflicts, raids or attempts to deport people,” summarizes a mother of 3 children in one of the recent case studies (Baron et al. 2019, p. 17). Everyday struggles of young people engendered by the isolated geographical position of the shelter and amplified by the scarcity of spaces that support learning and development within the shelter give way to the following questions: What are the activities supported by the environment in which these young people live? ; and what opportunities for cognitive and emotional growth are available to them within their sociocultural context?

We begin this inquiry into the situated experience of young people living in Berlin shelters by detailing barriers inherent in the current formal education system in Germany. We focus first on *Welcome Classes*, an educational approach practiced across the German school system, which has a specific aim to prepare non-German speaking students to enter mainstream classes. Furthermore, we describe limitations to participating in formal education that arise from living in shared accommodations, exacerbated by the Covid-19 pandemic and the resulting safety measures instituted to prevent the spread of the virus.

We then describe an emergency response carried out during the spring of 2020 at the shelter, designed as a deliberate theoretical intervention into processes of teaching and learning. This informal educational intervention, *digital homework mentorships*, aimed to support learning and social integration during a time of heightened isolation. Grounded in socio-cultural theory (Vygotsky 1978), the program intended not to replace formal education, but to supplement the schooling interrupted by the pandemic and resulting school closures. Furthermore, guided by the emerging conceptual framework of *pedagogies of care* (Motta and Bennett 2018; Beroš 2020), the program described here aimed at offering students support, structure, and a chance to make sense of the situation participants found themselves in. Following the conclusion of the intervention, our critical inquiry into the effectiveness of the program suggests that such one-on-one mentorships offer the inclusivity that is missing from Germany’s highly segregated schooling system. Insights can be drawn from the design, methods, and implementation of the program for future use in conjunction with more formal educational approaches specifically in situations where young people find themselves amidst the conditions of radical change.

## Germany’s “Welcome Classes”

According to official United Nations High Commissioner for Refugees (UNHCR) estimates, young people make up approximately 51% of all refugees worldwide. The UNHCR (2017) reported that in 2016 alone, approximately 1.3 million people came to Germany as refugees, 30% of them—or 400,000—under 18 years old. In the 2017/2018 school year, 1.2 million of 11 million, or 10.7% of students in Germany, were without a German passport (Destatis 2018).

All children in Germany not only have a right to education, but are required to attend school (Döbert 2007). Education institutions have the potential to counteract the isolation and segregation brought about by the use of special housing accommodations for refugees. But when students with refugee or migration backgrounds enter the school system, they are usually placed in separate classes, segregated from the majority student population. Schools are usually the first point of contact a refugee child has with the greater community and are therefore critically important in the process of adjusting to one’s new neighborhood (Kia-Keating and Ellis 2007). With so much in flux for children living in refugee shelters, school can provide a constant, fixed, and stable routine, ideally providing opportunities for social contacts and achievement, as well as the chance to begin constructing future endeavors (Zito and Martin 2016). On a psychological level, interpersonal interactions with peers and teachers at school can help students make sense and make meaning of their situated experience—especially during times of radical change (Lucić 2020). Research has also shown that fostering a sense of belonging at school can have significant positive effects on a child’s educational experience. Feeling more attached, committed, involved, and spirited about one’s school is directly linked to heightened self-efficacy among young refugees (Kia-Keating and Ellis 2007).

Despite clear signs since 2010 that Europe, and Germany especially, would most likely be confronted with large numbers of people seeking refuge within its borders, the education system was fully unprepared. In 2015 when German schools saw a large spike in children from asylum-seeking families wishing to be enrolled, there was no research, no plan, no examples of best practices, and no trained staff to be found (Schroeder 2018). One particularly worrying consequence of the improvised attempt to integrate non-German-speaking children into the education system is that they are typically separated from their local peers from day one of school (Karakayali et al. 2016). These separate classes, like the one Oduman attended for his first year of school, are commonly referred to as “Welcome Classes.”

Mona Massumi et al. (2015) distinguish five main categories for the various models of the classes or tracks into which non-German-speaking students are placed in Germany. They include the *submersion model* (*das submersive Modell*) in which new children are placed directly into mainstream classes together with their German speaking peers from the start, as well as the *integrated model* (*das integrative Modell*) where students are placed in a mainstream class, but receive additional German language lessons. In the *partially integrated model* (*das teilintegrative Modell*), students are in separate classes, but take part in mainstream classes in certain subjects. However, most commonly found (Schroeder 2018) is the *parallel model *(*das parallele Modell*) in which students are taught in separate classes until they are deemed ready to be matriculated into a mainstream class (Massumi et al. 2015). In the most segregated variant, the *parallel model with diploma* (*das parallele Modell Schulabschluss*), children remain in separate classes their entire school years, until they graduate (ibid).

Karakayali et al. (2016) studied a total of 18 classes containing students lacking German language skills in ten elementary schools across eight districts of Berlin. In five schools sampled, children from Welcome Classes attended lessons with children from mainstream classes a few hours per week, mostly in physical education and music class. Three of the schools followed an integrated approach, and in four schools, children were not taught together at all. The children were often separated physically, with some Welcome Classes in remote parts of the school building. In three of the schools sampled, the classes were held in rooms used for the afterschool program, which often led to conflicts surrounding who could use the rooms and when. In one school, the classes took place in a nearby shelter, so the children attending Welcome Class had virtually no contact with other students whatsoever. Generally, many teachers shared the perception that because Welcome Classes are so separate, children who attend them are often “forgotten,” left out of school-wide sporting events, first-day-of-school celebrations, school festivals, the assignment of timeslots for the gym, or from participating in theater pieces (ibid.).

In elementary schools where separate Welcome Classes are offered, there are usually two classes: One for children with ages corresponding to grades one through three and one for grades four through six. These classes have a high turnover rate. This is due to children entering the classes having just arrived in Germany, children moving out of an emergency shelter and having to switch schools, and children leaving class because they matriculated into mainstream classes. When children are deported, they disappear from the class overnight. These fluctuations, aside from being distressing to teachers and students, present huge challenges when it comes to creating and maintaining structure and group dynamics (Karakayali et al. 2016).

In the classes surveyed by Karakayali et al. 2016, no regular documentation with regard to a child’s academic progress took place. None of the schools had a concept for how children from Welcome Classes could gradually attend mainstream classes. When, and based on what criteria, a child would move from a Welcome Class to a mainstream class was not formally regulated either. Instead, each teacher decided how to handle this transition, usually by way of tests created by the teachers themselves. Although the teachers studied were generally highly motivated and invested in filling in the educational gaps their students presented, many of them were not qualified to teach children. None of the teachers in the sample had a certification to teach German as a foreign language, and few had formally studied education or pedagogy. The freshly employed Welcome Class teachers, new to the profession, received significantly less pay than the mainstream class teachers, worked on only limited contracts, and most complained of a lack of support for staff. Many of the teachers were reportedly interested in attending additional trainings, but due to lacking availability of substitute teachers, they were usually unable to (Karakayali et al. 2016).

Aside from massive organizational challenges, Welcome Classes carry with them a glaring blind spot. Schools are responsible not only for providing the resources to develop skills, but also for enabling social proficiency. The latter is as important for children with refugee and migration experience as it is for the rest of the student population. The question often raised is: If social proficiency includes getting along with culturally diverse people, how is it permissible to keep the two groups culturally isolated, apart from one another ? In 2016, the Berlin Ministry for Education, Youth, and Science (Senatsverwaltung für Bildung, Jugend und Wissenschaft) published a guideline for integration of new and migrant children and youth in childcare and schools (SenBJW 2016). The purported goal of Welcome Classes laid out in the guideline is students’ speedy acquisition of the German language in order to be matriculated into mainstream classes as quickly as possible (SenBJW 2016). However, teachers find this goal to be barely reachable precisely because students are kept apart from their German peers (Karakayali et al. 2016).

In effect, the practice of separation in schools places young people from immigrant backgrounds at a disadvantage, as cultural integration into German society is considered a minimal norm necessary for healthy adjustment and personal advancement in the society (Green 2004). On the social plane, as Bourdieu and Passeron (1977) show, advancement within a particular culture becomes a function of the familiarity of an individual with the culture of the host society and the dominant class. Hence, it is not surprising that German students are three times more likely than non-German students to be accepted to university (Korntheuer et al. 2018). Educational outcomes and inequities are not necessarily a result of what is taught or not taught at school, with regards to content. Rather, it is the notions alive in educational discourse that generate sustained marginalization and low expectations of certain groups of students (Ross, Dooly and Hartsmar 2012). Separate classes for newcomers are a practical ramification of this discourse, an exaggerated perception of difference and incompatibility that has resulted in exclusionary policy.

Zeus Leonardo (2002) warns “enslavement, discrimination, and marginalization of the Other work most efficiently when trolled, aggregated as the same” (p. 127). Welcome Classes are spaces for children from a multitude of cultures and ethnicities, crudely constructed as one group with common educational needs. Aside from the potential negative consequences on the self-concepts of the children directly affected by this grouping, for the children in mainstream classes, this process presents the Other as abstract, rather than concrete. With this comes a dangerous message, the subtext of which is the following: Diverse people from around the world are all the same (ibid.), and as such, all distinctly different from Germans.

## The Developmental Ramifications of Segregated Schooling

When discussing the *zone of proximal development*, Vygotsky (1978) emphasizes an interpersonal space where psychological development happens. Defined as the distance between the actual developmental level and the level of potential development achieved under adult guidance or in collaboration with a more capable peer—Vygotsky (1978) notes that “human learning presupposes a specific social nature and process by which children grow into the intellectual life of those around them” (p.86). As the brief discussion of Oduman’s case shows, the social, physical, and geographical position of refugees within a given society can greatly determine—allow, afford, or preclude—the radius of their developmental interactions. *Those around them* into who's intellectual life a child might grow into has traditionally been determined by the spatiotemporal position a developing person occupies. In other words, where a person lives largerly determines who they can interact, and develop, with. Interpersonal interactions across neighborhoods, schools, peer groups, and extracurricular activities are seen as contexts responsible for mediating and scaffolding the developmental process of an individual. Residing in geographically remote, socially isolated shelters while at the same time attending culturally segregated Welcome Classes, drastically limits the possibilities for interaction and development.

According to sociocultural theory, on the most basic level, cultural and environmental forces shape and render the development of the individual within any social context (Vygotsky 1978; Bronfenbrenner 1986; Cole 1996; Rogoff 2003). By growing up in a specific culture, individuals become familiar with the artifacts, roles, norms, and cultural practices that are unique to that culture. Human activity is in a dialectical relationship both with the culture and history of the particular society that the individual inhabits (Scribner 1985). Scholars have gone so far as to argue that even visual perception, and the cognitive patterns inherent in and affected by perception, are structured in response to the environmental context which surrounds the developing individual (Gibson 1979). Naturally, due to differences in culture, communal organization, educational systems, lifestyles, the level of economic development, and environmental context that exist between different societies—and often within societies—the guidance and developmental potential that a particular society provides to its members is unique and specific.

Migration to a new society is inherently a developmental process, which demands complex adjustments and requires changes on cognitive, intra-psychic, interpersonal, and social planes. During the process of migration, the culture within which development occurs changes radically. After arriving to a new country, migrants become exposed to a different sociocultural ethos, which often involves a new and culturally specific set of rules, roles, norms, and practices that they must follow. Upon entering a new society, migrants are also confronted with new sets of meanings, discourses, power-structures, spatial concepts, and even transportation routes which they need to follow in order to move forward—sometimes quite literally—both in the context of their new culture and in their personal development. Thus, migration disrupts an individual’s process of development within a specific culture. From the perspective of sociocultural theory, in order to function in their receiving culture, immigrants engage in activities, often discursive, that allow them to master and internalize the cultural tools of their host culture (Lucić 2013). Accordingly, cultural integration requires a number of changes and adaptations on cognitive, emotional, and interpersonal levels. Often, it requires migrants to transform some of their cultural practices and fuse them with those of their receiving society (Kwak 2003). This process becomes particularly difficult for individuals residing within predominantly immigrant housing projects, and those attending Welcome Classes, where there is little opportunity for interactions with the members of the host society.

Human activity, which is context specific, simultaneously shapes culture and is shaped by culture, along with cultural tools and interpersonal interactions that a given sociocultural context fosters. From this vantage point, every developmental activity is mediated through a set of context-situated interpersonal interactions. In other words, according to Vygotsky (1978), developmental processes are situated both in interactions with physical, cultural tools, such as books and smartphones, as well as in a more fluid set of interpersonal interactions with others—peers, teachers, adults—mediated through yet another cultural tool, language. The point of intersection among these concrete cultural tools and interpersonal interactions constitutes the *zone of proximal development* where, with assistance and in cooperation with the more capable peer, a child can reach a higher developmental level. In psychological development, as Vygotsky notes, “imitation and instruction play a major role. They bring out the specifically human qualities of the mind and lead the child to new developmental levels… What the child can do today in cooperation he can do alone tomorrow” (p.188). But given that young people from migrant backgrounds living in Germany are developing in largely segregated environments—and are educated in classes designed exclusively for newcomers—they are deprived of the opportunity to interact and cooperate with German peers. This not only prevents them from making sense of rules, roles, and norms in interpersonal interactions within the larger German society, it precludes them from engaging in developmentally beneficial interactions and cooperating with those who could naturally function as *more capable peers* with regards to the ways of being and interacting in Germany.

## Existing Inequalities Sharpened by the Onset of Covid-19 Pandemic

The problems confronted by people living in refugee shelters were exacerbated by the measures put in place to prevent the spread of Covid-19 in March 2020. While “staying at home” for many Berliners meant retreating to the safety of their apartments, people in often-crowded mass accommodations were exposed to various risks. The practice of keeping at least two meters apart in social interactions was largely impossible for residents in shelters.

For children, the lockdown had grave consequences. Regarding the interpersonal nature of learning, Vygotsky (1978) theorized that “what children can do with the assistance of others might be in some sense even more indicative of their mental development than what they can do alone” (p. 85). Before the lockdown brought about by the Covid-19 pandemic, young people at the shelter where Oduman lives could spend time with staff and volunteers—*more capable peers* with regards to the ways of being and interacting within their new social environment. These interactions happened in the playroom where free and organized play took place, or in the tutoring room where they could receive homework help. But with the onset of the pandemic the community rooms closed, and because it was impossible to ensure that physical distancing rules would be observed, young people were no longer allowed on playgrounds. Volunteers were not allowed to enter the camp and on-site staff was reduced. In effect, the protective factors in the form of leisure activities that used to provide stability—activities in the community rooms and access to outdoor space—disappeared. Without these supportive structures, young people in shelters have reported struggling with loneliness and suicidal thoughts (Baron et al. 2019).

With schools closed, young people who were already on the losing end of education inequality faced yet another wave of setbacks. For one, most children’s parents were unable to help their kids with schoolwork, as their German skills were typically poorer than their children’s. Schools in Berlin made use of online learning platforms, but access was limited for the children living at the shelter because there was rarely a direct line of communication between teachers and students or their parents (often because of language barriers and/or lack of familiarity with technology). Furthermore, many children did not have laptops on which they could make use of the online learning platforms and the Internet connection at the shelter was very weak. The attempts at the transition from in-person to virtual schooling has certainly shown and highlited that in the 21st century, everyone should be able to access the Internet. The introduction of technologically mediated teaching has virtually abolished free education for those without means to acquire a personal computer, or to access or pay for a reliable Internet connection. Hence, mobile phones—a tool largely ubiquitous among youth at the shelter—were identified as a potential avenue for maintaining communication while stay-at-home orders were in place.

## Homework Mentorships as an Answer to Education During the Lockdown

**Fig. 2:**
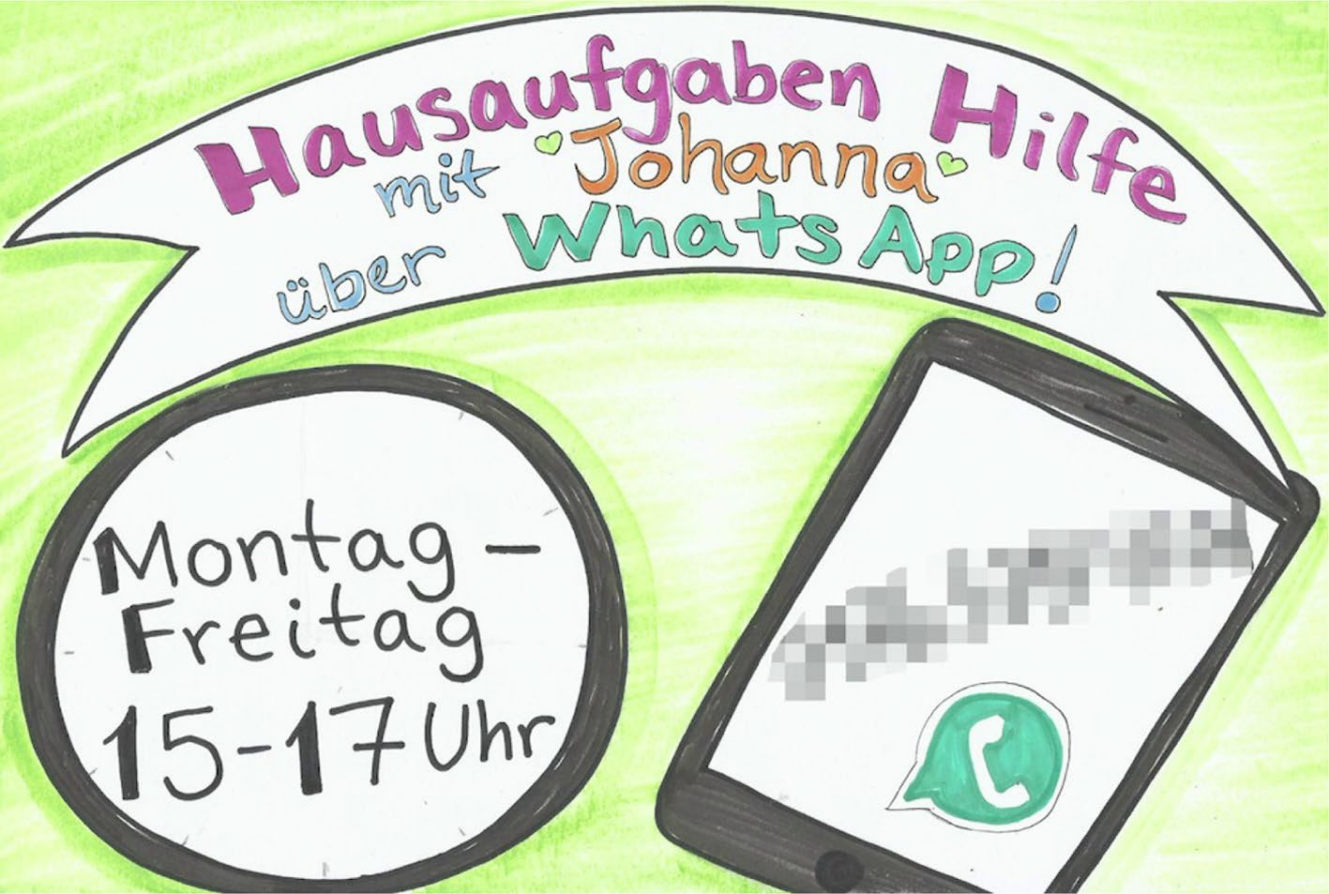
Flyer distributed to children and teens at the shelter at the start of school closures due to the lockdown in response to the Covid-19 pandemic.

In the beginning of April 2020, two child and youth counselors at Oduman’s shelter set up a homework help hotline for young residents. They made flyers with contact information on them and distributed them to the children (see Figure 2). Few called in though.

Through a conversation with an 11-year-old former resident of the shelter and her father, the concept of a more personal version of the homework hotline emerged: *Homework Mentorships*. Rather than leaving it to children (in many cases young) to repeatedly conjure up the initiative to call a hotline when they needed assistance, each student would be paired up with a native German-speaking adult who they could turn to for help. The same homework mentorship concept was introduced to two different volunteer groups working in Berlin. One of the groups focuses on promoting interaction among people with and without refugee and migration backgrounds, while the other consists of recent graduates from the Childhood Studies and Children’s Rights master’s course at the Potsdam University of Applied Science. In total, five people signed up to be mentors, joining the three staff members at the shelter interested in participating.

The methods used in the intervention included the following steps: Completing two online forms (a "needs assessment" and a “goals agreement” survey); regular communication between the mentor and mentee via phone calls and texting; weekly supervision (via Skype) with mentors; and the purchasing and presenting of motivational gifts upon achieving one’s homework goals. Before the end of the 2019/2020 school year in June, 16 children and teens received one-on-one homework help from a total of eight mentors via WhatsApp and Skype. Some mentor/mentee pairs continued to practice German on the phone during the summer holidays, while others continued to meet without a focus on homework. The mentees ranged in age from eight to 16 years old. Six participants attended  Welcome Classes in elementary as well as high schools, eight were in second, third, or fourth grade, and two had not yet started in a Welcome Class yet. Participants’ countries of origin included Afghanistan, Albania, Iran, Syria, Iraq, and Moldova.

As a first step to facilitate matching among mentors and potential mentees, a Google survey was created, entitled *Children’s Online Survey: Education without School?.* The goal of the survey was to assess the educational needs of the young people living at the shelter. All 14 to whom the survey was delivered responded. The survey’s first question asked if they understood what they were supposed to do for school, now that they were no longer attending school in person—ten responded that they did. Four reported that they did not understand what was expected of them from their teachers. The answers to the second, third, and fourth question indicated that the onset of the Covid-19 pandemic drastically reduced the contact respondents had with their teachers. Half had contact with their teachers only once per week; six had no contact with their teacher whatsoever; and only one person had contact with their teachers several times per week. Email was the form of communication used by half of the respondents who had contact with their teachers, followed by phone for three respondents and WhatsApp/Telegramm (both messaging apps) for two. The fifth question revealed that six children received what they assessed as *enough* schoolwork; five indicated that they received *too much* schoolwork; and three received *none at all*. Next, nine young people reported they could complete some of their school assignments on their own; four could complete none of their assignments on their own; and only one could complete them all on their own. The seventh question showed that nine students had a laptop, tablet, or mobile phone that they could use to do their assignments. Eleven respondents reported having a sufficient Internet connection to do their schoolwork. In the ninth question, respondents were asked if they had enough peace and quiet at home to focus on their schoolwork, to which nine out of sixteen responded in the affirmative. Next, children were asked if they would like to take part in the *Homework Help 2.0* program. They were explained that this meant they would receive homework help via WhatsApp and/or telephone calls when they needed it. All survey respondents said that they wanted to participate. Finally, respondents could write freely about anything in general they might need assistance with. Most respondents listed trouble with math or German language skills. One boy included that he could not understand many assignments without the instructions being explained to him. We later discovered that this was a common problem the children faced.

Some older children filled out the form themselves using their smartphones. One of the child and youth counselors also went to the camp, knocked on doors, and went through the questions with the rest of the young people in person. If their parents approved, their phone numbers were collected.

**Fig. 3:**
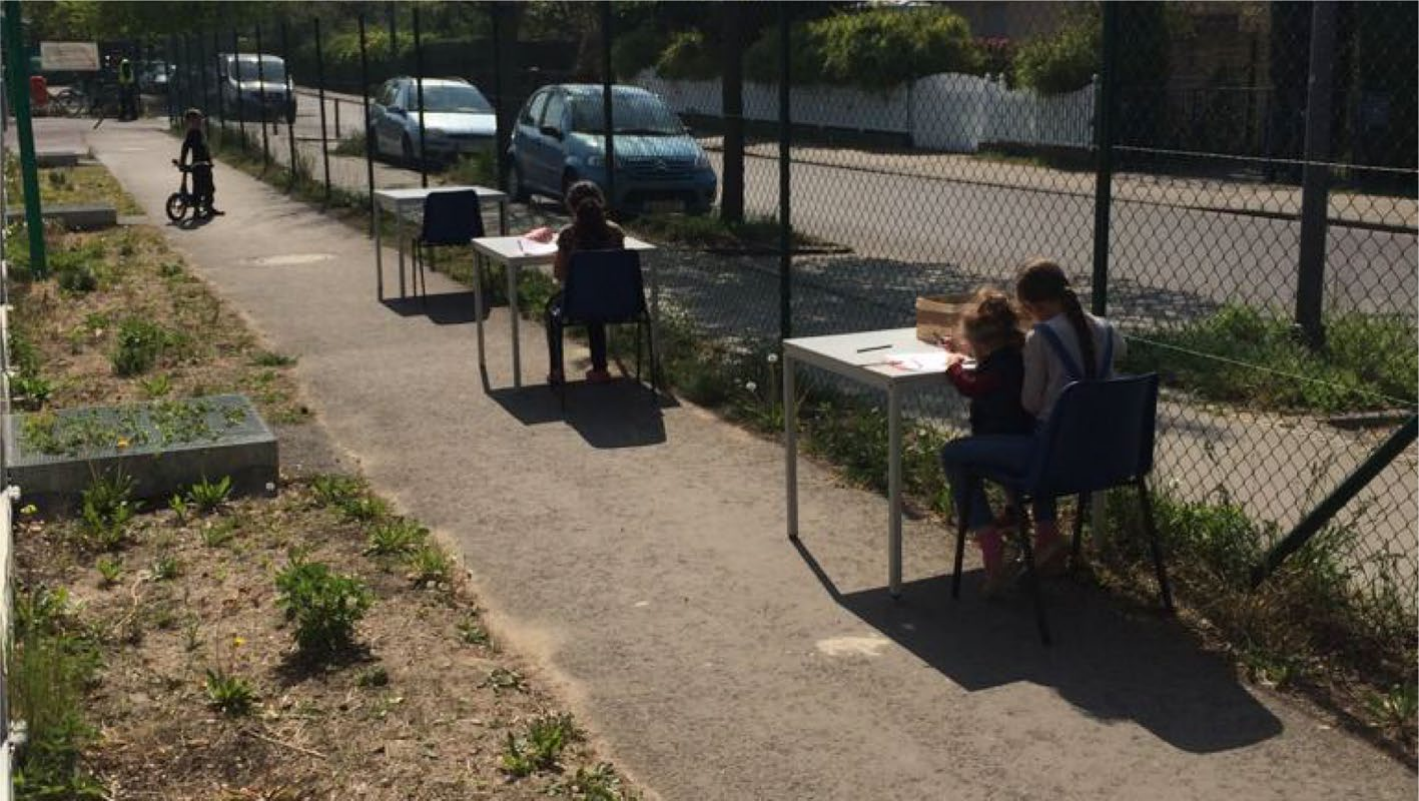
Children work on their homework just outside of the tutoring room, in compliance with the strict social distancing regulations in Berlin amid the initial outbreak of the Covid-19 pandemic.

For the initial conversation after mentees were matched to mentors, they received access to a second online form that was created by one of the counselors at the shelter. The aim of this survey was to set goals for the mentorship in light of the mentee's homeschooling pursuits. Questions on the form were about what assignments children still had to complete, how often and when exactly they wanted to speak on the phone, who should call whom, and what the mentee would like as a gift once they had completed their schoolwork. The involvement of motivational gifts created an opportunity for homework helpers to visit the camp and deliver the presents, sometimes meeting their mentees in person for the very first time. During the first month of the program, volunteers were not allowed inside the camp, but were able to take walks, visit nearby parks with their mentees, or come up with other creative solutions that were in compliance with the strict social distancing regulations (see Figure 3 for example).

## A Qualitative Evaluation of the Homework Mentorship Program

Before continuing with Oduman’s story, three other examples are presented here to provide a window into some of the processes that the mentorships contained. Despite the highly varied courses the different mentorships took, each case demonstrates an important element of sociocultural theory and the utility of approaching the questions of learning and development from this perspective. Specifically highlighted are the following theoretical aspects: The role of structured interpersonal interactions with more capable peers, the essential function of cultural tools in enabling the interpersonal interactions to occur, as well as the role of cultural tools in the development of higher psychological functions.

Tabea, a 31-year-old volunteer with a background in social work and children’s rights studies, worked with an 11-year-old Syrian boy attending a Welcome Class and a 9-year-old boy from Iran in the second grade. Both of them had been struggling in school—one with issues around attention and hyperactivity and the other with aggressivity towards his classmates. Neither of the boys knew Tabea previously. She was surprised to hear about their difficulties at school because they showed great enthusiasm, discipline, and enjoyment as they each spoke with her on the phone for 45 minutes per session, three times per week. The parents of the boy with attention issues reported that their son usually did not listen to them when they asked him to do his schoolwork, but was highly motivated to do so when Tabea called. This is consistent with Vygostky’s (1978) notion of zone of proximal development, which suggests that young people are highly responsive when developing interpersonal interactions—and learning with—more capable others who are closer in age. Structured interpersonal interactions with a mentor that allow the learner to observe and practice their learning skills can give way to intrapersonal growth. Once internalized, these interpesonal forms can lead to the development of higher psychological functions such as self-regulated learning. Even after the lockdown ended and schools resumed in-person lessons again during the last week of April 2020, the phone sessions continued to take place over the summer break and Tabea became a friend of the family, visiting them from time to time at the shelter. She planned to continue supporting the boy during the upcoming school year. In the case of the second boy, Tabea informed us that their calls ended abruptly. His caseworker was then able to follow up and address a situation that may have otherwise remained hidden. This incident highlights that the mentorships and the interpersonal interactions that the process engenders act as a sociocultural buffer and play a protective role. The lockdown meant that children were not seeing (or being seen by) teachers or caseworkers, making it all the more important that they had a line to the world outside of their small containers.

The developmental benefits of structured interpersonal interactions with peers are further highlighted by a case of a brother and sister residing at the shelter—9 and 10 years old—belonging to a Russian-speaking Roma family from Moldova. They were about to join a Welcome Class when the lockdown came. Because they spoke almost no German at all, they were often separate from the other children at the shelter. They were paired up with 30-year-old Judith, a volunteer who works in radio and has taught German to children and adults new to Berlin. The three of them could all speak Russian. Judith prepared worksheets for the children in Russian and German and held German lessons for them in Russian via live video. When Judith came to meet the family for the first time, a caseworker accompanied her, the children, and their mother to a park near the shelter. Judith coordinated with the Welcome Class teacher at a nearby elementary school and acquired workbooks for the two children. One of them worked diligently through the entire book with Judith’s support, and the other began speaking German rather confidently, playing regularly with the other children, who communicated with one another in German.

In addition to supporting the children’s learning process, the mentorship facilitated crucial processes regarding the physical health of the family. Only through Judith’s conversation in Russian with the children’s mother was it revealed that she had a heart condition. Judith communicated with the social workers at the shelter to arrange a doctor’s appointment for the mother, so that it could be determined if it was safe for the children to attend school once it reopened.

As mentioned earlier in this work, in addition to interpersonal interactions with more capable others like teachers and peers, according to Vygotsky (1978), developmental processes are situated both in interactions with physical, cultural tools such as books, articles, or smartphones. The utility of cultural tools is that they afford interpersonal interactions and enable higher psychological functions to emerge. This is specifically highlighted by Celina’s experience. She is a 21-year-old volunteer who is currently studying to become an elementary school teacher and first worked with a 16-year-old boy in a Welcome Class. He would send photos of assignments he needed help with and she would provide support via text or voice messages. After he no longer needed assistance, Celina was paired next with a 9-year-old Iraqi girl in a Welcome Class. She did not have access to a mobile phone, so staff at the shelter organized a donated one for her. Her father was, however, unable to register a SIM card for the phone because of his asylum status. Frustration was mounting and weeks passed without Celina and her mentee able to begin their homework collaboration, until Celina physically came to the shelter for the first time. She stood on the other side of the fence (visitors were not permitted to enter, due to restrictions to prevent the spread of Covid-19) and with the help of staff at the shelter was finally able to establish a line via Skype. They engaged in sessions over the phone, but soon Celina began picking her mentee up at the shelter every Friday to take her to the park around the corner where they practiced multiplication, did arts and crafts, and rollerbladed. When volunteers were able to enter the shelter again, Celina was there weekly and is planning to continue supporting the girl in the next school year.

Oduman had already had a mentor, Chris, who he began meeting with at the end of 2018. They built a robot together and Chris helped Oduman set up a Youtube channel where he posted videos of him playing his game of choice, Fortnite, along with humorous narration of the gameplay. By the time schools closed, Oduman was already spending all of his free time playing Fortnite. In general, he was almost never seen outside of his room and did not express any interest in taking part in activities offered by staff or volunteers at the shelter. Chris and Oduman’s caseworker were concerned about how the absence of school would affect Oduman. One Sunday afternoon when schools were completely closed, Oduman contacted Chris asking for his urgent help in completing a week’s worth of school assignments to be handed in the following day. Chris assisted him over the phone for several hours. Once the homework mentorship program was up and running, Oduman’s caseworker pitched to him the idea of signing up. He accepted the offer, providing Chris or Simeon, another staff member at the camp, was willing to be his mentor. Simeon was happy to take on Oduman, who became the 16th participant in the program. Oduman chose to phone daily to work on his assignments. It was revealed that he had just completed his first “big book” and loved it. For the remainder of the summer, he received support from Simeon for his academic pursuits and maintained his relationship with Chris, who continued teaching him skills for his successful Youtube channel, a strong source of motivation and pride for Oduman.

## Discussion: Inclusive Schools and Meaningful Personal Relationships

The homework mentorships were a response to an emergency situation, but they underscored shortcomings of the current formal education system in Germany and point to a way that informal learning can help fill in the gaps. The classroom is generally where a developing mind meets the cultural context of their larger society. On a psychological level, schools are the context where young people have the possibility of engaging in interpersonal interactions with culturally diverse others, leading to development of socio-cognitive complexity—the capacity to construe people, objects, and ideas in culturally appropriate, multidimensional ways (Lucić and Liharska 2019). However, as we have attempted to show, education offered to young people with refugee or migration backgrounds within the German school system often fosters exclusion. Given that the process of migration and subsequent social integration can be a bewildering experience even for well-educated adults, for young people, this process can be extremely challenging. Adolescence in general is associated with anxiety, but “culturally subordinate” students face additional pressure at school (Nieto 1999, p. 179). If their native languages and cultures are assigned a low status in the societies they live in, they are also subject to that society’s low expectations of them and may find they are completely unrepresented in traditional curricula. Sonia Nieto (1999) believes schools and schooling practices can actually produce low self-esteem in children, arguing that “students do not simply develop poor self-concepts out of the blue; rather, their self-esteem in terms of schooling is the result of policies and practices in schools that respect and affirm some groups while devaluing and rejecting others” (p. 195). Schools need to make room for students to define themselves, offering support as children grow into changing identities and create for themselves autonomous life-worlds. Schools have the potential to be nurturing spaces, where varied and fluid realities are not just tolerated or included, but truly appreciated (Schleimer 2018).

Integrated or submersion models of Welcome Classes promote contact among students with various backgrounds. Indeed, the three schools surveyed by Karakayali et al. (2016) that followed an integrated program saw success. In these schools, children with refugee and migrant backgrounds were included in all aspects of school life. Children were placed into a mainstream class according to their age and typically received additional German lessons. These schools faced far fewer organizational problems. Students with refugee or migrant experience in these classes, like their local peers, received instruction from trained elementary school teachers, as well as extra German lessons from people with training in teaching German as a foreign language. From the start of their school experience in Germany, children in these schools received a comprehensive curriculum including a range of subjects, while in the company of the other children in mainstream classes. Employing this approach, the transition is complete when additional German instruction is no longer needed. The native-German-speaking students in these classes also had the opportunity to get to know the new children in their communities, and get acquanited with some of their cultural practices.

Creating a nation-wide system for Welcome Classes based on best practices taken from schools who chose to use submersion or integrated models, rather than the common parallel model, could give newcomers such as Oduman a chance to get an education without being seen by their peers—and by themselves—as naturally existing outside of the mainstream. After all, how can Germany reduce its class divide if children learn at school that foreigners belong in a separate class? Additionally, as the examples of the homework mentorship program show, paying direct and individualized attention to the needs of newely arrived children can start to bridge the cultural divide.

Even if schools are reformed and become spaces where children of all backgrounds can feel empowered and included, the lives of many children on the move will remain volatile. In Oduman’s case, if his family does not manage to find an apartment by the time their current shelter closes at the end of 2020, they will be moved into different temporary accommodations. For Oduman, this would most likely mean new teachers, new social workers, and new neighbors. Germany dedicates abundant financial resources, programs, initiatives, and social services to integration of refugees and migrants. What seems to have actually helped Oduman were personal relationships such as the ones contained within the homework mentorship program. These relationships have the potential to remain intact, no matter where he is moved to next. Mentors such as the ones described in this work are able to offer assistance with actual school assignments, but they can also fill communication gaps between teachers, social workers, and parents, acting as sort of education managers for children. A meaningful bond with a supportive adult familiar with the host society provides avenues to academic, as well as social proficiency. But perhaps most importantly, it creates continuity when the key aspects such as living accommodations, educational institutions, and professionals to which a child has access to, are regularly changing.

The Covid-19 pandemic presented a state of crisis affecting people across socio-economic circumstances. But, we must not forget that people living in shelters are living in a prolonged state of crisis. Informal learning and personal relationships, such as the ones described in this work, have the potential to create crucial conditions of care and support that may be absent from formal institutions, no matter how turbulent times are.
